# Natural History of Atherosclerosis and Abdominal Aortic Intima-Media Thickness: Rationale, Evidence, and Best Practice for Detection of Atherosclerosis in the Young

**DOI:** 10.3390/jcm8081201

**Published:** 2019-08-12

**Authors:** Michael R. Skilton, David S. Celermajer, Erich Cosmi, Fatima Crispi, Samuel S. Gidding, Olli T. Raitakari, Elaine M. Urbina

**Affiliations:** 1 Boden Collaboration for Obesity, Nutrition, Exercise and Eating Disorders, Charles Perkins Centre, The University of Sydney, Sydney 2006, Australia; 2 Sydney Medical School, Faculty of Medicine and Health, The University of Sydney, Sydney 2006, Australia; 3 Department of Woman’s and Child’s Health, Obstetrics and Gynecology Clinic, University of Padua, 35128 Padova, Italy; 4 BCNatal—Barcelona Center for Maternal-Fetal and Neonatal Medicine (Hospital Clínic and Hospital Sant Joan de Deu), 08028 Barcelona, Spain; 5 Institut d’Investigacions Biomèdiques August Pi i Sunyer, Universitat de Barcelona, 08036 Barcelona, Spain; 6 Centre for Biomedical Research on Rare Diseases (CIBER-ER), 28029 Madrid, Spain; 7 FH Foundation, Pasadena, CA 91106, USA; 8 Centre for Population Health Research, University of Turku and Turku University Hospital, FIN-20520 Turku, Finland; 9 Research Centre of Applied and Preventive Cardiovascular Medicine, University of Turku, FIN-20520 Turku, Finland; 10 Department of Clinical Physiology and Nuclear Medicine, Turku University Hospital, FIN-20520 Turku, Finland; 11 Heart Institute, Cincinnati Children’s Hospital, University of Cincinnati, Cincinnati, OH 45229, USA

**Keywords:** atherosclerosis, ultrasound, intima-media thickness, aorta

## Abstract

Atherosclerosis underlies most myocardial infarctions and ischemic strokes. The timing of onset and the rate of progression of atherosclerosis differ between individuals and among arterial sites. Physical manifestations of atherosclerosis may begin in early life, particularly in the abdominal aorta. Measurement of the abdominal aortic intima-media thickness by external ultrasound is a non-invasive methodology for quantifying the extent and severity of early atherosclerosis in children, adolescents, and young adults. This review provides an evidence-based rationale for the assessment of abdominal aortic intima-media thickness—particularly as an age-appropriate methodology for studying the natural history of atherosclerosis in the young in comparison to other methodologies—establishes best practice methods for assessing abdominal aortic intima-media thickness, and identifies key gaps in the literature, including those that will identify the clinical relevance of this measure.

## 1. Rationale for Assessing Aortic Atherosclerosis

### 1.1. Natural History of Atherosclerosis

Atherosclerosis is a disease process that is characterized by the build-up of fats, cholesterol, calcium, and other substances in the arterial wall, resulting in arterial wall thickening and the development of arterial plaques. This progressive pathophysiologic process begins early in life, but typically only becomes symptomatic in adulthood. It is the major disease process that underlies myocardial infarction and ischemic stroke.

The natural history of atherosclerosis in the major arteries, particularly the coronary arteries and the aorta, has been studied for over a century [1,2,3]. Key aspects that inform the theoretical advantages and disadvantages of abdominal aortic intima-media thickness (IMT), compared to other methodologies, include the timing of onset of adaptive intimal thickening and early lesions, macroscopic and microscopic phenotyping, the progression of lesions, and lesion-prone sites. 

Adaptive intimal thickening is a physiologic response to variations in blood flow and wall tension. This thickening is most pronounced at sites prone to the development of atherosclerotic lesions, particularly at or near arterial bifurcations and branches, including the internal carotid (carotid sinus) and the coronary arteries, and along the dorsal wall of the abdominal aorta [1]. Straight non-branching sections of the arterial vasculature, such as the common carotid artery, can develop a more diffuse adaptive intimal thickening, albeit one that is less severe [4]. Sites with adaptive intimal thickening express cellular and functional alterations prior to the development of microscopic lesions, including a small number of macrophages, increases in low density lipoprotein particles, and a turnover of endothelial and smooth muscle cells [1,5,6]. 

The earliest lesions are characterized by the accumulation of macrophage foam cells. These type I microscopic lesions can occur at the same sites that are prone to adaptive intimal thickening, and are perhaps most marked on the dorsal wall of the abdominal aorta where they are ubiquitous in the fetus and throughout early childhood [7,8]. 

The earliest macroscopic lesions are fatty streaks, which occur predominantly at the same sites as adaptive intimal thickening and microscopic lesions. These type II lesions first develop in infancy, with up to 50% of infants having visible fatty deposits in the aorta, and become quasi-ubiquitous by early childhood [9,10,11]. 

The onset of fatty streaks in the coronary arteries is slightly delayed relative to the aorta, not occurring until mid-childhood [10,12]. In the International Atherosclerosis Project, a large multi-ethnic study with over 23,000 sets of dissected coronary arteries and aortas collected between 1960–1965 from 14 countries in the Americas (North, Central, and South America), Caribbean, Europe, Africa and Asia, the extent of fatty streaks in the abdominal and thoracic aortas was greater than that in the coronary circulation at all ages from 10 through 69 years [12]. An autopsy study of children and young adults nested within the Bogalusa Heart Study, a long-term epidemiological study in a rural biracial population in Louisiana USA (since 1973), found that the extent of fatty streaks (percent surface involvement) was moderately correlated between the aorta and coronary arteries (Spearman correlation = 0.45) [13], indicating that aortic atherosclerosis in the young may be a suitable proxy for coronary atherosclerosis. 

In the internal carotid artery, there is no fatty deposition during the perinatal period, followed by a gradual increase from two years of age, such that by age 16 years, approximately 40% of children exhibit evidence of lipid deposition [14]. For the common carotid artery, fatty deposits first appear during early adolescence [15], with fatty streaks being present just proximal to the carotid bifurcation in nearly all individuals by early adulthood [16]. The prevalence of fatty streaks is lower in the more proximal segments of the common carotid artery [16].

These early lesions, which are non-occlusive, occur at the same sites at which intermediate and advanced lesions subsequently develop, and there is some evidence to indicate the direct progression of early lesions to intermediate lesions and advanced lesions [17,18]. In the International Atherosclerosis Project, the age of onset of raised lesions was similar in the coronary circulation and abdominal aorta (26 and 27 years, respectively), although the extent of disease progressed faster in the abdominal aorta (involvement of 0.90% of abdominal aortic intimal surface per year versus 0.55% of coronary intimal surface per year) [12]. In the Bogalusa Heart Study, the prevalence of fibrous plaques was greater in the aorta than in the coronary arteries in children (2 to 15 years), but lesser in each subsequent age group at 26–39 years [11]. The extent of fibrous plaques was only moderately correlated between the aorta and coronary artery (Spearman correlation = 0.31) [13]. In an autopsy study from Denmark undertaken between 1996–1999, the prevalence of raised lesions in the left anterior descending artery increased throughout adulthood from 12% at 20–29 years to about 50% at 30–39 years, and to 86% at 70–79 years [19]. The prevalence of raised lesions was slightly higher in the carotid bifurcation (at the carotid bulb and proximal internal carotid; about 20% of individuals aged 20–29 years, 60% aged 30–39 years, finally reaching over 80% of individuals at 60–69 years), but lower in the common carotid artery proximal to the bifurcation (0% at 20–29 years, 12% at 30–39 years, 66% at 60–69 years) [19].

### 1.2. Abdominal Aortic Atherosclerosis, Cardiovascular Risk Factors, and Cardiovascular Events

In addition to age-related progression, the extent and severity of atherosclerotic lesions in the abdominal aorta is also associated with cardiovascular risk factors. In adolescents and young adults, the extent of lesions in the aorta and coronary arteries is associated with established cardiovascular risk factors, including hypertension, obesity, and low density lipoprotein cholesterol levels, and inversely with high density lipoprotein cholesterol levels [11,20]. In fetuses and children, the extent and severity of microscopic lesions in the abdominal aorta is associated with age and maternal cholesterol levels, and inversely with birth weight [7,8]. Tobacco exposure is more strongly related to abdominal aortic atherosclerosis than coronary atherosclerosis in young adults [21].

Furthermore, abdominal aortic wall thickness by magnetic resonance imaging (MRI) is associated with risk of incident cardiac and vascular events [22]. This is consistent with the strong associations of abdominal aortic atherosclerosis with coronary and carotid atherosclerosis in late adolescence and young adults [21], and in turn, the substantial body of evidence linking coronary and carotid atherosclerosis in adulthood with incidences of clinical cardiovascular events [23].

## 2. Abdominal Aortic IMT as a Marker of Preclinical Atherosclerosis in Children and Adolescents

As outlined above, the dorsal wall of the abdominal aorta is more prone to the early development of fatty streaks and raised lesions than either the internal or common carotid arteries. As such, it was initially proposed that abdominal aortic IMT “might provide a better index of preclinical atherosclerosis in high-risk children than carotid IMT” [24]. Indeed, children and adolescents with cardiovascular risk factors have more pronounced abdominal aortic intima-medial thickening than carotid intima-medial thickening (Table S2) [24,25,26], and more pronounced favorable differences in abdominal aortic IMT than common carotid IMT in the presence of putative cardio-protective lifestyle factors [27,28]. Consistent findings have been observed for both mean and maximal measures of carotid IMT, and for measures of carotid IMT that focus solely on the common carotid as well as those that average both internal, bulb, and common carotid IMT measures [25].

Aortic IMT may also be an age-appropriate methodology in high-risk fetuses and infants [29,30]. As discussed earlier, although microscopic and macroscopic lesions are present, the structural determinants of aortic wall thickening in these very early age groups likely involve adaptive intimal thickening and inflammation. In contrast, in early adulthood, the assessment of carotid IMT may be more important. In the Muscatine Offspring study (*n* = 635), the PDAY risk score was more strongly associated with abdominal aortic IMT than with carotid IMT in adolescents (11 to 17 years), whereas the inverse was true for young adults (18 to 34 years) [25]. 

Abdominal aortic IMT by ultrasound has been directly validated. In post mortem samples from male adults, ex vivo assessment of abdominal aortic IMT by ultrasound is tightly correlated with direct measures from pathology [31]. In the fetus, histology of the abdominal aorta from a single growth restricted stillborn (33 weeks’ gestation) fetus was compared with samples from a non-growth-restricted fetus [32]. The growth-restricted fetus had evidence of abdominal aortic intima-medial thickening detected by both ultrasound and histology, altered elastin structure, macrophage infiltration, and endothelial cell activation, none of which were present in the abdominal aorta of the non-growth-restricted fetus [32]. 

### Association of Aortic IMT with Cardiovascular Risk Factors and Response to Interventions

**Figure 1:**
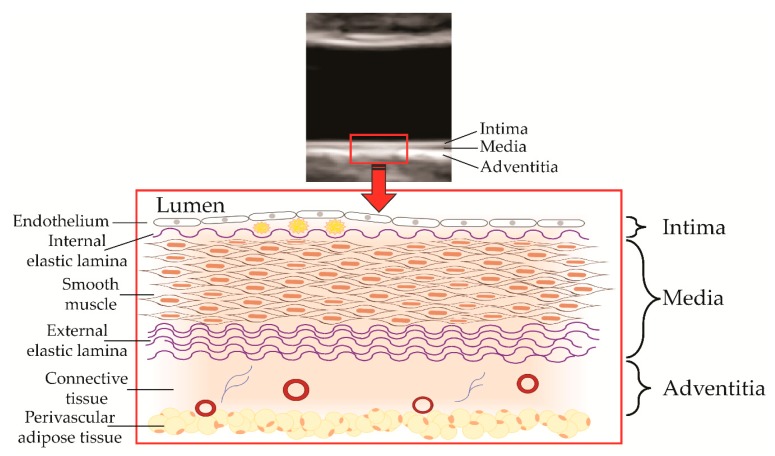
Components of the arterial wall as captured by ultrasound, including the tunica intima with macrophage foam cells in the subendothelial space, the tunica media, and the tunica adventitia consisting of connective tissue, fibroblasts, vasa vasorum, and nerves.

**Figure 2:**
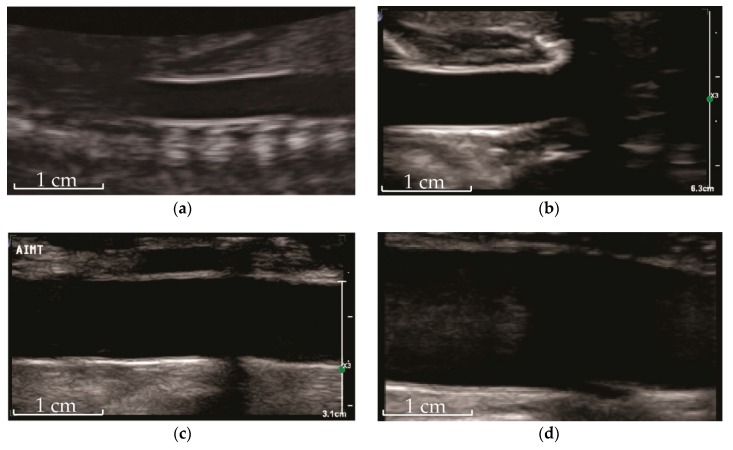
Ultrasound of abdominal aorta showing intima-media complex in a (**a**) fetus, (**b**) newborn, (**c**) child (8 years), and (**d**) young adult (20 years). Indicative scale as indicated.

The measurement of abdominal aortic IMT by high-resolution ultrasound (Figure 1) has enabled the non-invasive study of emerging and established cardiovascular risk factors on the extent and severity of atherosclerosis in infants, children, and adolescents (Figure 2 and Table S1). Findings for some of these emerging risk factors, such as impaired fetal growth, are supported by experimental work in animals, clinical studies in adults using carotid IMT, epidemiologic associations with cardiovascular events, and Mendelian randomization studies, which support causality [33,34,35,36]. 

**Figure 3:**
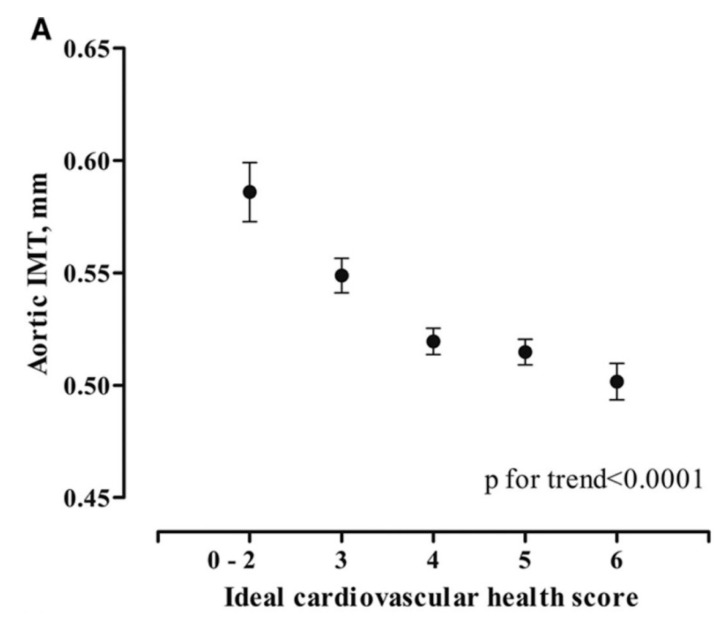
Ideal cardiovascular health in adolescence and abdominal aortic IMT. Ideal cardiovascular health score is the sum of the following health behaviors and factors: never smoked a cigarette; body mass index (BMI) <85th percentile; >60 min of moderate-intensity activity per day; four to five components of a healthy diet (fruit and vegetables ≥450 g/d, fish ≥200 g/wk, whole-grain bread ≥2 oz (28 g)/d, sodium <1500 mg/d, sugar-sweetened beverages ≤450 kcal/wk); total cholesterol <4.4 mmol/L; blood pressure <90th percentile; plasma glucose <5.6 mmol/L [38]. Reproduced with permission from Pahkala, K., Hietalampi, H., Laitinen, T.T., Viikari, J.S.A., Rönnemaa, T., Niinikoski, H., Lagström, H., Talvia, S., Jula, A., Heinonen, O.J., et al., Ideal Cardiovascular Health in Adolescence; published by Circulation, 2013.

Assessment of abdominal aortic IMT may also provide insight into putative interventions, particularly those relevant to children and adolescents. For example, leisure-time physical activity and respiratory fitness are favorably associated with abdominal aortic IMT in 17-year-olds [27,37]; a moderate increase in physical activity during adolescence is associated with reduced progression of abdominal aortic IMT [37], the maintenance of a healthy cardiovascular risk profile is strongly associated with lower abdominal aortic IMT throughout adolescence (Figure 3) [38], and dietary intake of short-chain omega-3 fatty acids is associated with lower abdominal aortic IMT in late adolescence in those born small for gestational age [28]. These potentially beneficial associations are now beginning to be tested in prospective randomized trials with abdominal aortic IMT as a prespecified outcome (e.g., ACTRN12616000053426). 

## 3. Methodological Considerations for Abdominal Aortic IMT

Detailed best practice guidelines for abdominal aortic IMT assessment are provided in Appendix A. These best practice guidelines include scanning protocols, equipment, and measurement. They have been formulated with a focus on promoting methodology consistent with the greatest feasibility and reproducibility. The current evidence indicates that participant characteristics are important contributors to feasibility and reproducibility of the technique (Table S3). Specifically, abdominal aortic IMT is least feasible in people with obesity (approximately 85% successful acquisition, compared to ≥97% successful acquisition in healthy weight and overweight), and has the lowest reproducibility in young adults (22% coefficient of variation for repeated scans, compared to 9% for adolescents). Adherence to these proposed best practice guidelines will facilitate a comparison of research outcomes and assist in achieving research priorities.

## 4. Assessment of Subclinical Atherosclerosis: Strengths and Limitations of Current Techniques

### 4.1. Carotid IMT

The assessment of carotid IMT by high-resolution B-mode ultrasound is an established methodology that reflects the burden of subclinical atherosclerosis. The evidence base to support the assessment of carotid IMT in adults, and methodological considerations, are described in detail elsewhere [39,40,41]. 

In vitro assessment validation by histology indicates that carotid intima thickness tends to be overestimated by ultrasound, and media thickness tends to be underestimated [42]; however, the thickness of combined intima and media is accurately assessed [31,42]. Accuracy is similar when comparing ultrasound measures with in situ pressure fixed samples [31]. 

Carotid IMT increases with age, by approximately 0.003 to 0.004 mm per year in adolescence and 0.012 to 0.017 mm per year in adulthood (mean carotid IMT) [43,44]. Furthermore, carotid IMT in adults is associated with established and emerging cardiovascular risk factors; these are higher in males, those with high blood pressure, obesity, diabetes, and those who smoke, among other factors [25,45,46], and responds to lifestyle, pharmacologic, and other interventions [44,47,48,49,50]. Importantly, carotid IMT assessed in adulthood is associated with the risk of incident myocardial infarction and stroke, independent of established risk factors [23], although the ability of carotid IMT to improve the classification of risk of cardiovascular disease is small at best [51,52]. Furthermore, although individual studies with few events suggest that the progression of carotid IMT over time predicts cardiovascular events [53], a meta-analysis with a large sample size (but combining studies with a variety of techniques and reproducibility) found no relationship [54]. 

Associations of risk factors with carotid IMT, and the response to interventions, have also been described in childhood [40,55,56]. However, the natural history of atherosclerosis in the carotid artery, in particular the emergence of fatty streaks in the common carotid artery only during the second and third decades of life [15], casts doubt on the usefulness of common carotid IMT in young children as a measure of atherosclerosis. A recent study of 280 children aged between 1–15 years found that carotid IMT was similar from 1–10 years of age, and increased only thereafter [57]. Furthermore, a number of studies have demonstrated that associations of risk factors with carotid IMT emerge at around 8–12 years of age. The International Childhood Cardiovascular Cohorts Consortium, which includes the Bogalusa Heart Study and Muscatine Study among others, found that carotid IMT in adulthood was only associated with childhood cardiovascular risk score when the risk score was derived from risk factors assessed at 9 years of age or older [58]. Children with familial hypercholesterolemia have higher carotid IMT from 12 years onwards, when compared to their unaffected siblings [59]. Finally, a systematic review found that adiposity does not appear to be associated with carotid IMT in children less than 12 years, concluding that the “thickening of the carotid artery with adiposity may only become detectable in later childhood and adolescence” [60]. In contrast, the assessment of carotid IMT during infancy has been limited, although a single study found that impaired fetal growth is associated with higher common carotid IMT during infancy [61], which is consistent with an established body of evidence in adults [34,62]. Given the absence of lesions in the carotid artery during infancy, this raises the possibility that IMT may at least partially reflect non-atherosclerotic mechanisms. Nonetheless, the majority of studies showing associations of risk factors with carotid IMT in children have focused on those 8 years or older [24,56,63,64,65,66,67,68,69]. The age from which variance in—and progression of—carotid IMT begins to reflect atherosclerotic disease processes, and subsequently predict future atherosclerotic vascular disease events, should be the focus of future research, particularly given the ability to longitudinally track carotid IMT through to later adulthood.

### 4.2. Coronary Artery Calcium

Use of this tool is typically discouraged in childhood because coronary calcification does not generally occur until the fourth decade of life, atherosclerosis can be present in the absence of calcification in young individuals, and there is radiation exposure. Pediatric studies are thus far limited to familial hypercholesterolemia where coronary artery calcium has been shown to be prevalent in 28% of adolescents [70].

### 4.3. Magnetic Resonance: Carotid and Aortic Wall Thickness

MRI can be used to measure aortic wall thickness. It is non-invasive, without radiation exposure, and thus can be used safely in otherwise healthy individuals, including potentially children and adolescents. However, MRI is considerably costlier, and equipment is less readily available than for ultrasound. 

Arterial wall thickness measured by 1.5T MRI appears to comprise the entire wall (intima, media, and adventitia) [71]. In adults, abdominal aortic wall thickness by MRI predicts future cardiovascular events [22] and the lifetime predicted risk of cardiovascular disease [72]. Thoracic aortic wall thickness is associated with individual cardiovascular risk factors, including age, male gender, body mass index, and blood pressure [73,74]. Abdominal and thoracic aortic wall thicknesses by MRI are not increased in adolescents with type 1 diabetes and high cholesterol compared to healthy controls, although the thoracic wall had greater irregularity in those with diabetes [75]. Atheroma have been seen in adolescence in the abdominal aorta in the setting of extreme dyslipidemia [76]. Future studies may seek to directly compare aortic IMT by ultrasound and aortic wall thickness by MRI, in the same participants, to potentially enable long-term tracking of aortic atherosclerosis from childhood through late adulthood, including in people with obesity. 

### 4.4. Pulse Wave Velocity (PWV)

PWV is a widely applied and accepted marker of arterial stiffness. There are several distinct methodologies employed to assess PWV. The most widely published methodology, and that with the strongest evidence base, is derived from the transit time and distance between the carotid and femoral arteries, consisting predominantly of pulse transit along aorta. The evidence base to support the assessment of pulse wave velocity in adults, and methodological considerations, are described in detail elsewhere [77,78]. 

The main vascular properties that affect PWV along the aorta in adults are distinct from those that drive arterial IMT. Aortic PWV is mainly a structural measure that is principally related to the maintenance of elastin fibers and degradation thereof with aging, and the deposition of collagen fibers. Other arterial properties that affect arterial stiffness include heart rate and endothelial function, although the latter is of lesser importance in the aorta than it is in more muscular arteries [78]. As such, PWV is determined by the structural and functional properties of the aorta, and is distinct to those that drive abdominal aortic IMT. 

Aortic PWV is associated with established cardiovascular risk factors, perhaps most strongly with hypertensive disorders and aging. The direction of causality between hypertension and arterial stiffness is controversial [78], whereas aging may at least partly act as a surrogate for the total duration of exposure to risk factors.

In childhood and adolescence, risk factors for higher aortic PWV include sex, age, and height, in addition to body mass index, blood pressure, heart rate, dyslipidemia, and impaired fetal growth [78,79,80,81,82,83,84]. However, the age at which aortic PWV begins to increase is not clear, with some proposing that the first pronounced increase occurs at around 10–12 years of age [85].

## 5. Abdominal Aortic IMT: Gaps in Knowledge and Research Priorities

**Figure 4:**
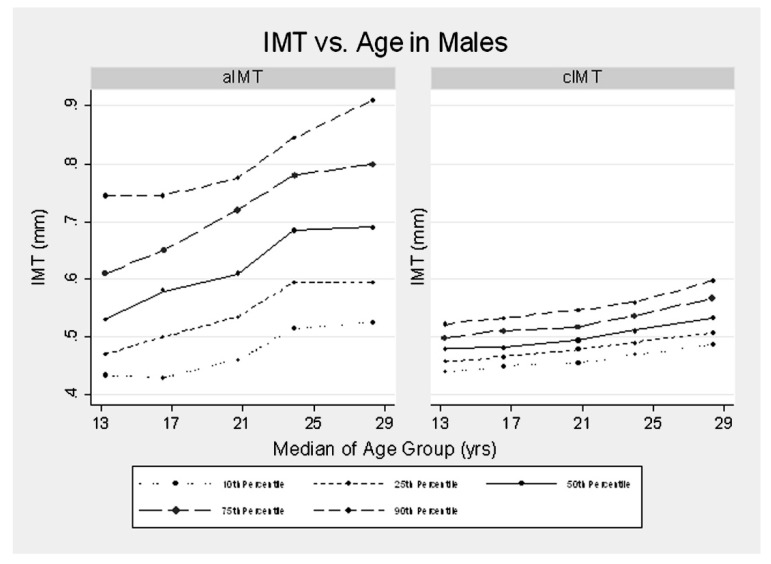
Percentiles of abdominal aortic intima-media thickness (IMT) and carotid IMT according to age in males. Female trends were similar but slightly lower (0.03 mm less for abdominal aortic IMT and 0.02 less for carotid IMT). The mean increase in abdominal aortic IMT with age was 0.10 mm/decade as compared to 0.04 mm/decade for carotid IMT [86]. Reproduced with permission from Davis, P.H., Dawson, J.D., Blecha, M.B., Mastbergen, R.K., Sonka, M., Measurement of Aortic Intimal-Medial Thickness in Adolescents and Young Adults, published by Ultrasound in Medicine & Biology, 2010.

Key gaps in current knowledge and research priorities are given in Table S3, including comparison and validation with histology, the development of normative data across the life course (Figure 4), detailed feasibility and reproducibility at different ages and in different body sizes, longitudinal tracking and ascertainment of normal rates of progression, and trialing abdominal aortic IMT for utility in clinical practice, including in comparison with other risk markers and measures of vascular health. Potential clinical uses that warrant study include for assessing treatment benefit (e.g., to monitor atherosclerotic disease progression in children with familial hypercholesterolemia and to monitor their individual response to lipid-lowering therapies), and risk stratification (e.g., screening for at-risk individuals amongst the offspring of adults identified with severely premature cardiovascular disease, yet without overt cardiovascular risk factors).

## 6. Summary and Conclusions: Abdominal Aortic IMT and Associated Methodologies to Further the Study of the Natural History of Atherosclerosis

Pathologic studies demonstrate that the earliest phases of atherosclerosis result in the vascular thickening of susceptible large and medium caliber vessels, making technologies that assess vascular thickness and stiffness feasible tools to study atherosclerosis development. Regions of the aorta are impacted earliest in development.

- Aortic and carotid atherosclerosis.
  - In children less than 8 years of age, the abdominal aorta is the site with the most pronounced early atherosclerosis, and where the strongest associations of risk factors with arterial IMT are observed.
  - In children and adolescents aged 8 to 12 years, associations of risk factors with arterial IMT are observed in the abdominal aorta and the carotid arteries. The association of risk factors with carotid IMT is the most pronounced in high-risk groups (e.g., diabetes and chronic kidney disease) [87,88].
  - In adolescents and young adults, obesity is an important factor limiting the feasibility of abdominal aortic IMT assessment. MRI holds promise as a research tool for the accurate assessment of both abdominal aortic wall thickness and stiffness in this age group, although further research is required.
  - Longitudinal studies tracking abdominal aortic and carotid atherosclerosis from fetal life through adulthood are required.
- Arterial stiffness. The assessment of pulse wave velocity should be considered as a measure of complementary pathophysiological processes, particularly in those aged ≥10 years.
- Multiple methodologies should be considered, particularly in adolescence, for more complete profiling of atherosclerosis development throughout the arterial tree early in the life course.

The abdominal aortic IMT methodology as described in this paper provides a template for the appropriate evidence-based implementation and reporting of this technique in research. We have identified several gaps in the literature. Addressing these will inform the clinical relevance of abdominal aortic IMT and advance our understanding of the natural history of atherosclerosis. 

## Supplementary Materials

The following are available online at https://www.mdpi.com/2077-0383/8/8/1201/s1, Table S1: Established, emerging and novel cardiovascular risk factors & aortic IMT, Table S2: Effect size comparison: risk factors and IMT, both abdominal aortic and carotid, Table S3: Key gaps in current knowledge and proposed research priorities.

## Appendix A. Best Practice Guidelines for Abdominal Aortic IMT Assessment

These best practice guidelines are summarized in Table A1.

### Appendix A.1. Scanning Protocol—Location

Assessment of thoracic aorta IMT is possible using transesophageal echocardiography in adults [89], although the invasive nature of transesophageal echocardiography restricts its use in healthy children and adults.

The majority of studies assessing aortic IMT by non-invasive ultrasound in children and young adults have imaged the abdominal aorta with the probe positioned on the ventral surface. Pregnancy offers a unique opportunity to obtain coronal or sagittal views of the fetal aorta positioning the probe on the maternal abdomen through the amniotic fluid cavity.

In fetuses, infants, children, and adults, the abdominal aorta is the preferred site for the assessment of aortic IMT, given that (a) post mortem studies indicate that the extent and severity of lesions in early childhood are greatest in the arch and abdominal aorta [7,8]; (b) the severity of lesions in the abdominal aorta continues to progress during early childhood in the abdominal aorta of high-risk individuals [7]; and (c) the ultrasonic window for scanning the abdominal aorta in children and adolescents consists primarily of soft tissue, as compared to that for the aortic arch, which is complicated by the presence of the thoracic cage.

The selection of the exact portion of the abdominal aorta for assessment of aortic IMT may be based on (a) image quality, (b) an anatomical landmark (e.g., just proximal to bifurcation) [24], or (c) predisposition of the site to formation of atheroma. For the latter, the distal, dorsal surface of the abdominal aorta is a site at particular risk of advanced lesion development [90].

**Table A1:** Summary of best practice guidelines for abdominal aortic intima-media thickness (IMT).

Aspect	Best Practice Guidelines for Abdominal Aortic IMT Assessment
Scanning protocol - Location	- Abdominal aorta. - Exact section determined by (a) image quality, (b) an anatomical landmark (e.g., just proximal to bifurcation), or (c) predisposition of the site to formation of atheroma. - IMT measured from far wall. - Study protocol should prospectively state the prespecified site, or criteria for site selection if based on image quality.
Scanning protocol - Imaging	- Abdominal aorta imaged in longitudinal section, horizontal on screen. - Images should use an appropriate zoom.
Equipment	- Linear array probe (≥7 MHz) is preferred. - For multi-frequency probes, settings that favor the frequency spectrum ≥7 MHz should be prioritized. - Lower imaging frequencies may be required in fetuses, adults, and when scanning at greater depth. - Ultrasound equipment and settings, including frequency, dynamic range, and persistence, should be standardized in each research study and maintained throughout.
Loop acquisition	- Minimum of one digital loop, with ≥3 cardiac cycles.
Measurement and analysis	- Report both mean and maximum abdominal aortic IMT. - Mean abdominal aortic IMT should be assessed in a region free of plaques. - Measurement of abdominal aortic IMT should be undertaken at end-diastole. - Semi-automated measurement of abdominal aortic IMT with dedicated software is preferred to measurement with calipers. - Measurement using semi-automated analysis derived from a minimum of 4 mm of abdominal aortic wall, over a minimum of 3 cardiac cycles. - Blinded image analysis. - Measurement methodology should be applied consistently. - Multi-center studies should use a single center for all IMT measurements. - Reproducibility of imaging and measurement should be undertaken periodically, and published.
Longitudinal studies (cohorts and trials)	- All scanning and assessment methodology should remain consistent across multiple visits, where possible. - Methods that promote the analysis of aortic IMT at the same arterial region should be applied. - Studies with serial measures of abdominal aortic IMT should have a minimum total follow-up of 12 months, but preferably 24 months or longer. - In addition to baseline and final visit abdominal aortic IMT, scans should also preferentially be obtained at an intermediate time-point(s).
Statistical analysis	- Results should be presented with and without adjustment for body habitus or abdominal aortic diameter, preferably by inclusion as a covariate. - In studies with sufficient statistical power, consideration should be given to dichotomizing abdominal aortic IMT into “elevated” and “normal” groups, using the age-specific, sex-specific, and population-specific 95th percentile to identify those with “elevated” abdominal aortic IMT in childhood and adolescence.
Feasibility	- Feasibility of technique in a specific group, defined as a successful measurement in a minimum of 90% of all participants (arbitrary cut point).

The scan site may still be restricted by the availability of suitable ultrasonic windows, which may differ by age. For example, gas in the abdomen of newborns restricts the available window to image the abdominal aorta just proximal to the bifurcation. In this group, a more readily available window is situated between the umbilicus and diaphragm. The prespecified site, or criteria for site selection if based on image quality, should be clearly stated in the study protocol or manuscript, especially for longitudinal studies. The fasting status may influence bowel gas, and as such, age-appropriate participant fasting should be considered in the study design as a potential means to maximize the available ultrasonic window. In the prenatal period, the amniotic fluid surrounding the fetus permits imaging the fetal aorta not only in the abdominal portion, but the aortic arch and/or thoracic aorta may be feasible also.

The far wall of the aorta is currently the preferred site for analysis, as opposed to the near wall. For an ultrasonic assessment of carotid IMT, comparisons with histology demonstrate the far wall IMT to be a more accurate reflection of the combined intima and media thickness than the near wall [42]. Nonetheless, using a composite measure that incorporates both the near and far wall carotid IMT has been shown to be of benefit in risk prediction. Future research may seek to determine whether there are similar benefits for the incorporation of near-wall abdominal aortic IMT.

### Appendix A.2. Scanning Protocol—Imaging

The aorta should be maintained in a horizontal position on screen, in a longitudinal section perpendicular to the ultrasound beam, to maximize the resolution. Images should use an appropriate zoom. Ultrasound equipment with a write zoom should preferentially be used to maximize the spatial resolution in the zoomed region, as opposed to a read zoom, which purely magnifies the unzoomed pixels.

If the scan site is selected to be relative to a specific landmark (e.g., bifurcation), that landmark should be visible in the image to enable accurate location assessment.

### Appendix A.3. Equipment

Assessment of abdominal aortic IMT has previously been undertaken with both portable and mainframe ultrasound machines. Portable ultrasound machines are potentially attractive for assessing abdominal aortic IMT in the research environment given their generally lower cost and flexibility for use in diverse environments (e.g., maternity wards). Limitations of portable ultrasound machines typically include lower processing power and poorer image quality than top-end mainframe machines.

A ≥7 MHz linear array probe should preferentially be used for imaging abdominal aortic IMT, as per arterial IMT validation studies [42]. The assessment of arterial IMT using a curved, multi-plane, vector, phased, or non-linear array transducer has not been appropriately validated. Curved array probes are an attractive alternative, given their availability, image quality—particularly at depth—and their ergonomic comfort. A direct comparison of relative and absolute measures from linear array and curved probes would provide important de facto validation of curved probes for abdominal aortic IMT assessment. Sonographer comfort is an important aspect determining scan quality, and further validation of curved array probes, and further investigation of the reproducibility and validity of abdominal aortic IMT when using such is warranted [86]. Lower-frequency transducers provide greater tissue penetration, and have been used to assess abdominal aortic IMT in fetuses, adults, and when required for greater scanning depth [30,86,91]. Nonetheless, transducers with frequencies <7 MHz have not been appropriately validated for the assessment of IMT. For multi-frequency probes or those that image across a range of frequencies that extend below 7 MHz (e.g., a 5–12 MHz probe), settings that favor the frequency spectrum ≥7 MHz should be prioritized.

The same brand/model ultrasound machine and transducer should be used for all the scans within each study. All the machine settings, including frequency, dynamic range, and persistence, should be standardized and maintained throughout the entirety of each research study.

For multi-site trials in which the equipment available differs between sites, ultrasound settings should be standardized, as closely as possible, across all sites. Nonetheless, care should be taken when interpreting differences in abdominal aortic IMT between sites, given that this may relate to differences in equipment. A repeatability study comparing the different machines should be considered as part of the study protocol.

### Appendix A.4. Loop Acquisition

For each participant visit, at least one digital loop consisting of at least three cardiac cycles should be captured for offline analysis. Digital file compression should be turned off or minimized.

### Appendix A.5. Radio Frequency Analysis

An assessment of IMT directly from the radio frequency (RF) signal has greater spatial resolution than that from an assessment of a visually-represented B mode ultrasound signal [92]. Thus far, there are no publications that report abdominal aortic IMT assessed directly from the RF signal. Current algorithms for assessing arterial IMT from the RF signal were developed for the assessment of carotid IMT, and may not robustly detect the abdominal aortic IMT in practice (personal communication, Skilton). An assessment of abdominal aortic IMT from the RF signal would theoretically increase the precision and reduce the variance of the measure, and thus would potentially be of particular relevance in longitudinal applications involving repeated measures, such as clinical trials, and in any future application detailing the response to treatment in individual patients. The development of algorithms that assess abdominal aortic IMT directly from the RF signal should be a priority for future research.

### Appendix A.6. Measurement/Analysis

A measure of mean thickness and a measure of maximal thickness should both be reported. Measures of mean thickness and maximum thickness likely reflect different aspects of the pathophysiology of early subclinical atherosclerotic lesions, particularly relating to the extent and severity of lesions, respectively. Lesion severity is theoretically more relevant from adolescence onwards, when raised macroscopic lesions are present. In younger children, the pathophysiology of differences in abdominal aortic IMT are less clear, as previously described.

Measurement of abdominal aortic IMT should be undertaken at end-diastole, given evidence of changes in intima-medial thickness during the cardiac cycle for carotid IMT. Currently, no such published evidence exists of changes in aortic IMT throughout the cardiac cycle, nor whether the extent of any such aortic intima-medial compression relates to vessel caliber, IMT itself, or risk factors. The end-diastole can be determined by electrocardiography, or by concurrent assessment of the change in vessel diameter, the latter of which is particularly relevant in the assessment of abdominal aortic IMT in fetuses and infants.

Abdominal aortic IMT can be assessed manually with calipers or semi-automatically with software that measures the IMT at each point along a segment delineated by a region of interest. Manual sonographic caliper measurements of abdominal aortic IMT have been shown to be lower than those measured by semi-automated edge-detection methods [93], although this will likely differ based on subjective aspects of the manual measurement protocol. The relative advantages and disadvantages of these two measurement approaches when applied to carotid IMT are described in detail elsewhere [94,95]. In brief, semi-automated methodologies capture more data, are less operator-dependent, and are less subjective than caliper-based methods. The assessment of abdominal aortic IMT by semi-automated edge-detection software has higher repeatability and reproducibility than that assessment by calipers [86,93].

Two points of distinction between the assessment of abdominal aortic IMT and carotid IMT are the shorter length of the vessel from which abdominal aortic IMT can generally be assessed, and the shorter duration of the loop length, which is often obtained from younger, less compliant participants.

For semi-automated measures, we propose that the region of interest for IMT measurement should include a minimum length of abdominal aortic wall of 4 mm over a minimum of three cardiac cycles, as a likely reasonable balance between time constraints and acceptable vascular coverage.

Analysis should be undertaken offline by an observer blinded to participant characteristics. Consistent measurement methodology should be applied in a given study. In multi-center studies, a central “IMT reading lab” should analyze all the images to maximize consistency.

All groups should assess the reproducibility of their measurement technique on abdominal aortic IMT images prior to commencing measures for their initial study. Reproducibility should be re-assessed periodically, approximately every three years. In multi-center studies, reproducibility should be reported for each site. Reproducibility findings should be published and reported using widely accepted methodologies.

### Appendix A.7. Longitudinal Studies (Cohorts and Trials)

All scanning and assessment methodology should remain consistent across multiple visits, and methods that promote the analysis of aortic IMT at the same arterial region should be applied. In situations where physical characteristics necessitate a different scan site (section of abdominal aorta) at different study visits, care should be taken when interpreting the absolute progression of abdominal aortic IMT.

Studies with serial measures of abdominal aortic IMT should have a minimum total follow-up of 12 months, but preferably 24 months or longer.

In addition to baseline and final visit abdominal aortic IMT, scans should also preferentially be obtained at an intermediate time point(s)—for example, at six months for a trial of 12 months duration, or every six to 12 months for trials of two years or more. Cohorts with intentions to follow-up over a longer time period may choose to have less frequent scans, focusing on those at critical age periods (e.g., infancy, pre-school, pre-adolescence, late adolescence, and young adulthood).

### Appendix A.8. Statistical Analysis

Results should be presented both with and without adjustment for a measure of body habitus or abdominal aortic diameter, assessed concurrently at the same site as abdominal aortic IMT [96]. Adjustment should preferably be made by including one of these measures as a covariate [96]. For infants, care should be taken in the interpretation of the abdominal aortic IMT to birth weight ratio (if used). Whilst attractive and intuitive, the resulting variable will be inversely proportional to birth weight. The use of this ratio in groups of participants with a similar birth weight may be warranted [97], although results are less clear when used in participant groups with widely divergent birth weights [29]. For body size, it would be preferable that the measure used is independent of adiposity.

In studies with sufficient statistical power, consideration should be given to dichotomizing abdominal aortic IMT into “elevated” and “normal” groups. Study-specific cut points will provide certainty for a priori power calculations, but the use of cut points based on age-specific and sex-specific normative values will enable a ready comparison of findings from different studies. The application of such normative values is currently limited due to the effect on absolute abdominal aortic IMT measurements of equipment and measurement protocol. As such, the adoption of normative values will be dependent on uniform methodology for abdominal aortic IMT assessment (e.g., equipment and measurement protocol), and potentially the machine-specific calibration of algorithms.

There is currently no direct evidence that a particular threshold in childhood and adolescence will identify those at increased risk of adult cardiovascular disease events. We recommend the use of the age-specific, sex-specific, and population-specific 95th percentile as a cut point to identify those with “elevated” abdominal aortic IMT in childhood and adolescence. This is based on the following: approximately ~10% of young adults (age 32 to 46 years) have a high-risk vascular phenotype (coronary artery calcium), and are at increased risk of cardiovascular disease events [98]; there is a lack of evidence for a specific cut point in childhood and adolescence to identify long-term cardiovascular risk.

Less conservative cut points may be required in smaller studies to ensure sufficient statistical power; however, when doing such, care should be taken to avoid the use of terms such as “elevated IMT”. Such terms may be seen to imply pathology, which in the absence of longitudinal data linking early abdominal aortic IMT with later cardiovascular events, would unnecessarily label a group of young people that likely consists of an important proportion with “normal” arterial wall thickness and non-elevated risk.

### Appendix A.9. Feasibility

For the methodology to be considered feasible in a given study, we propose that abdominal aortic IMT should be measurable in a minimum of 90% of all participants, including those who are not cooperative with the scanning protocol (e.g., unsettled newborns). This is a relatively arbitrary level; albeit, it is consistent with the body of work currently available for abdominal aortic IMT. Specifically, current evidence suggests that abdominal aortic IMT assessment can be highly feasible in fetuses [30], infants [99], including newborns [29], adolescents, and young adults [25,38], based on successful measurement in ~95% of participants, and also appears feasible in middle-aged adults with the use of a lower frequency transducer [91,100]. There is a lack of evidence concerning the feasibility and usefulness of the technique in the elderly. Body size, particularly the target depth (from skin surface to the far wall of the aorta along the plane of insonation) is a key driver of feasibility [86], due to short wavelength (high-frequency) ultrasound not being able to penetrate deeply [101]. In a protocol that allowed for the use of a lower frequency curved array transducer when the abdominal aorta was visualized at 7 cm or greater depth, abdominal aortic IMT was least feasible in adolescent participants with a BMI above the 95th percentile and adult participants with a BMI above 30 kg/m^2^ [86].

The assessment of aortic IMT in the fetus has been demonstrated between 30–36 weeks gestation [30,102], although imaging may be possible as early as 20–26 weeks’ gestation (Cosmi and Skilton, personal communications).
