# Supplementary material for: Natural History of Atherosclerosis and Abdominal Aortic Intima-Media Thickness: Rationale, Evidence, and Best Practice for Detection of Atherosclerosis in the Young
Source: J Clin Med. 2019 Aug 12;8(8):1201. doi: 10.3390/jcm8081201 (PMC6723244; doi:10.3390/jcm8081201)
Supplement: Supplementary file 1 [file jcm-08-01201-s001.pdf]

SUPPLEMENTAL MATERIAL

Natural History of Atherosclerosis & Abdominal Aortic Intima-Media Thickness: Rationale, Evidence and Best Practice for Detection of Atherosclerosis in the Young.

Michael R. Skilton, David S. Celermajer, Erich Cosmi, Fatima Crispi, Samuel S. Gidding, Olli T. Raitakari, Elaine M. Urbina.

SUPPLEMENTARY TABLES

Table S1. Established, emerging and novel cardiovascular risk factors & abdominal aortic IMT.

Exposure	Participants	Effect size
**Type 1 diabetes** [1]<br><br><br><br>**Type 1 diabetes** [2]	Children at 11 y (SD 2) with:<br>Type 1 diabetes (n=44)<br>*vs.* healthy children (n=28)<br><br>Children at 14.1 y (SD 2.5) with:<br>Type 1 diabetes (n=66)<br>*vs.* healthy children (n=32)	Mean abdominal aortic IMT<br>0.50 (0.09)<br>*vs.* 0.44 (0.05)<br><br>Mean abdominal aortic IMT<br>0.57 (0.11)<br>*vs.* 0.50 (0.01)<br>Maximum abdominal aortic IMT<br>0.69 (0.14)<br>*vs.* 0.61 (0.09)
**Hypercholesterolemia** [1]	Children at 11 y (SD 3) with:<br>Hypercholesterolemia (n=16)<br>*vs.* healthy children (n=28)	Mean abdominal aortic IMT<br>0.53 (0.10)<br>*vs.* 0.44 (0.05)
**Tobacco smoke exposure** [3]	Children at 13 y with serum cotinine at 8-13 y: low exposure (n=159),<br>*vs.* intermediate exposure (n=167)<br>*vs.* high exposure (n=161)	Maximum abdominal aortic IMT<br>0.527 (0.113)<br>*vs.* 0.563 (0.139)<br>*vs.* 0.567 (0.126)
**Impaired fetal growth** [4]<br><br><br><br><br><br><br><br>**Impaired fetal growth** [5]<br><br><br><br><br><br><br><br>**Impaired fetal growth** [6] *	Newborns at 2 days (SD 1) of age with birth weight:<br><10th percentile (n=24)<br>*vs.* 50-90th percentile (n=23)<br><br>Newborns on day 5 with birth weight:<br><10th percentile (n=40)<br>*vs.* 50-90th percentile (n=40)<br><br>Fetuses at 32 weeks' gestation (range 30-34) and early childhood at 18 months (range 17-21) with estimated fetal weight:<br><10th percentile & abnormal Doppler velocimetry (n=38 fetuses; n=22 early childhood)<br>*vs.* 10-90th percentile & normal Doppler velocimetry (n=32 fetusus; n=25 early childhood)<br><br>Fetuses at 34 weeks' gestation (range 32-35) with estimated fetal weight:	Mean abdominal aortic IMT<br>558 µm (59)<br>*vs.* 534 µm (58)<br>Maximum abdominal aortic IMT<br>810 µm (113)<br>*vs.* 743 µm (76)<br><br>Mean abdominal aortic IMT<br>0.52 (0.03)<br>*vs.* 0.40 (0.03)<br>Maximum abdominal aortic IMT<br>0.58 (0.06)<br>*vs.* 0.44 (0.05)<br><br>Fetus:<br>Mean abdominal aortic IMT<br>1.9 (1.35-2.37)<br>*vs.* 1.15 (0.95-1.43)<br><br>Early childhood:<br>Mean abdominal aortic IMT<br>2.4 (1.5-3.1)<br>*vs.* 1.03 (0.88-1.24)<br><br>Mean abdominal aortic IMT<br>0.504 (0.477, 0.530)

Table S1. Established, emerging and novel cardiovascular risk factors & abdominal aortic IMT.

Exposure	Participants	Effect size
**Impaired fetal growth** [7] †	<10th percentile & abnormal Doppler velocimetry (n=35)<br>*vs.* <10th percentile & normal Doppler velocimetry (n=40)<br>*vs.* 10-90th percentile & normal Doppler velocimetry (n=49)	*vs.* 0.466 (0.477, 0.485)<br>*vs.* 0.471 (0.454, 0.488)
**Impaired fetal growth** [8] ‡	Newborns (during first week) with birth weight:<br><3rd percentile or abnormal Doppler velocimetry (n=35),<br>3-10th percentile & normal Doppler velocimetry (n=32),<br>*relative to control group with birth weight >10th percentile & normal Doppler velocimetry (n=134)*	Mean abdominal aortic IMT<br>0.036 (SE 0.018) in severe SGA,<br>0.023 (SE 0.017) in modest SGA,<br>*both relative to control.*<br>Maximum abdominal aortic IMT<br>0.057 (SE 0.022) in severe SGA,<br>0.055 (SE 0.018) in modest SGA,<br>*both relative to control.*
**Impaired fetal growth** [9]	Infants at 6 months corrected age with birth weight:<br><10th percentile (n=100)<br>*vs.* 10-90th percentile (n=32)	Mean abdominal aortic IMT<br>0.569 (0.065)<br>*vs.* 0.485 (0.066)<br>Maximum abdominal aortic IMT<br>0.564 (0.071)<br>*vs.* 0.665 (0.070)
**High birth weight** [10] §	Newborns at 3-5 days with:<br>birth weight >90th percentile (n=30)<br>*vs.* appropriate birth weight for gestational age newborns (n=30)	Mean abdominal aortic IMT<br>0.49 (0.03)<br>*vs.* 0.39 (0.03)
**Birth weight** [11] †	Infants at 6.2 (SD 1.5) weeks of age participating in a population-derived prebirth cohort (n=835)	Mean abdominal aortic IMT<br>20.6 µm (13.7, 27.6) per kg birth weight<br>Maximum abdominal aortic IMT<br>28.4 µm (19.0, 37.9) per kg birth weight
**Gestational diabetes** [10] §	Newborns at 3-5 days of age of mothers with diabetes mellitus and birth weight >90th percentile (n=40)<br>*vs.* birth weight >90th percentile alone (n=30)	Mean abdominal aortic IMT<br>0.56 (0.06)<br>*vs.* 0.49 (0.03)
**Perinatal microbial exposure** [12] †	Infants at 6.2 (SD 1.5) weeks of age participating in a population-derived prebirth cohort (n=757); 21% with maternal group B streptococcus colonization in third trimester	Mean abdominal aortic IMT<br>14.1 µm (5.6, 23.0) higher in offspring of women with group B streptococcus colonization

Table S1. Established, emerging and novel cardiovascular risk factors & abdominal aortic IMT.

Exposure	Participants	Effect size
**Persistent *Chlamydia pneumonia* seropositivity** [13]	Children at 11 years of age participating in long-term lifestyle trial (STRIP, since 6-months of age; n=128). *Chlamydia pneumonia* seropositivity annually from 7-11 years:<br>persistent seropositivity (25%),<br>*vs.* transient seropositivity (18%),<br>*vs.* seronegative (57%)	Mean abdominal aortic IMT<br>persistent 0.532 (0.086)<br>*vs.* transient 0.494 (0.061)<br>*vs.* seronegative 0.496 (0.054)
**Early infancy adiposity** [11] †	Infants at 6.2 (SD 1.5) weeks of age participating in a population-derived prebirth cohort (n=789); mean neonatal skinfold thickness (triceps and subscapular)	Mean abdominal aortic IMT<br>4.6 µm (1.4, 7.9) per mm skinfold thickness<br>Maximum abdominal aortic IMT<br>5.9 µm (1.5, 10.2) per mm skinfold thickness
**Early infant weight gain** [11] †	Infants at 6.2 (SD 1.5) weeks of age participating in a population-derived prebirth cohort (n=821); weight change from birth to 6 weeks	Mean abdominal aortic IMT<br>11.8 µm (3.7, 24.8) per kg weight change<br>Maximum abdominal aortic IMT<br>13.8 µm (2.8, 24.8) per kg weight change
**Maternal obesity** [14] †	Newborns at 0-7 days of age of women with first trimester:<br>overweight or obesity (n=9)<br>*vs.* healthy weight (n=23)	Maximum abdominal aortic IMT<br>0.07 mm (0.01, 0.12) higher in offspring of women with overweight or obesity

Results reported as mean and SD; except * median (range), † mean (95% CI), ‡ mean (SE), § mean (not specified). Units are mm; unless otherwise indicated.

Table S2. Effect size comparison: risk factors and IMT, both abdominal aortic and carotid, during childhood and adolescence.

Age & Exposure	Effect size: abdominal aortic IMT	Effect size: carotid IMT
**Newborns**<br>Impaired fetal growth [8] *	Mean abdominal aortic IMT<br>0.036 (SE 0.018) in severe SGA,<br>0.023 (SE 0.017) in modest SGA,<br>*both relative to control.*<br><br>Maximum abdominal aortic IMT<br>0.057 (SE 0.022) in severe SGA,<br>0.055 (SE 0.018) in modest SGA,<br>*both relative to control.*	Mean common carotid IMT<br>0.023 (SE 0.010) in severe SGA,<br>0.017 (SE 0.008) in modest SGA,<br>*both relative to control.*<br><br>Maximum common carotid IMT<br>0.026 (SE 0.013) in severe SGA,<br>0.035 (SE 0.011) in modest SGA,<br>*both relative to control.*
**Age 11 y (2)**<br>Type 1 diabetes [1]	Mean abdominal aortic IMT<br>0.50 (0.09)<br>*vs.* 0.44 (0.05) in control group	Mean common carotid IMT<br>0.47 (0.04)<br>*vs.* 0.42 (0.04) in control group
**Age 11 y (3)**<br>Hypercholesterolemia [1]	Mean abdominal aortic IMT<br>0.53 (0.10)<br>*vs.* 0.44 (0.05) in control group	Mean common carotid IMT<br>0.46 (0.04)<br>*vs.* 0.42 (0.04) in control group
**Age 11 y**<br>Persistent *Chlamydia pneumonia* seropositivity [13]	Mean abdominal aortic IMT<br>persistent 0.532 (0.086)<br>*vs.* transient 0.494 (0.061)<br>*vs.* seronegative 0.496 (0.054)	Mean common carotid IMT<br>persistent 0.439 (0.051)<br>*vs.* transient 0.431 (0.034)<br>*vs.* seronegative 0.444 (0.043)
**Age 13 y**<br>Tobacco smoke exposure [3]	Maximum abdominal aortic IMT<br>0.567 (0.126) high<br>*vs.* 0.563 (0.139) intermediate<br>*vs.* 0.527 (0.113) low	Maximum common carotid IMT<br>0.535 (0.006) high<br>*vs.* 0.525 (0.005) intermediate<br>*vs.* 0.502 (0.006) low
**Age 14.1 y (2.5)**<br>Type 1 diabetes [2]	Mean abdominal aortic IMT<br>0.57 (0.11)<br>*vs.* 0.50 (0.07) in control group<br><br>Maximum abdominal aortic IMT<br>0.69 (0.14)<br>*vs.* 0.61 (0.09) in control group	Mean common carotid IMT<br>0.43 (0.05)<br>*vs.* 0.42 (0.05) in control group<br><br>Maximum common carotid IMT<br>0.51 (0.06)<br>*vs.* 0.50 (0.06) in control group

Results reported as mean and SD; except * mean (SE). Units are mm; unless otherwise indicated.

Table S3. Key gaps in current knowledge and proposed research priorities.

Key knowledge gaps & rationale for priority	Current evidence	Priorities & potential methods
**Comparison & validation with histology.**<br><br>Inform the relationship of abdominal aortic IMT with wall thickness, composition, inflammation, smooth muscle cell hyperplasia, extracellular matrix alterations, and pathophysiologic processes consistent with atherosclerosis.	Fetus: histology of the abdominal aorta from a growth restricted stillborn fetus (33 weeks' gestation), indicated abdominal aortic intima-medial thickening (both ultrasound and histology), altered elastin structure, macrophage infiltration (presence of CD68), and endothelial cell activation (e-selectin); none of which were present in the aorta of a non-growth restricted fetus [15].<br><br>Adults: *post mortem* samples from male adults, assessment of abdominal aortic IMT *ex vivo* by ultrasound is correlated with histology and gross pathology [16].	*Post mortem* studies with greater sample size.<br><br>Animal models.
**Normative data.**<br><br>Enable identification of elevated abdominal aortic IMT, and comparison thereof across populations.<br><br>Implications for clinical utility.	Fetus (30-34 weeks' gestation): mean abdominal aortic IMT 1.15 mm (range 0.95-1.43)[6]. *Measured manually by sonographic caliper, in 32 fetuses with estimated fetal weight (10-90th percentile) and normal feto-placental Doppler velocimetry.**<br><br>Fetus (32-35 weeks' gestation): mean abdominal aortic IMT 0.471 mm [7]. *Measured in 49 fetuses with estimated fetal weight (10-90th percentile) and normal Doppler velocimetry*.<br><br>Newborns: mean abdominal aortic IMT 0.51 mm (SD 0.041)[17]. *Measured manually by sonographic caliper, in 100 healthy birth weight (50-90th percentile) term newborns from pregnancies not affected by hypercholesterolemia, diabetes, or history of maternal smoking.*<br><br>Newborns: mean abdominal aortic IMT 0.385 mm (SD 0.019)[18]. *Measured manually by sonographic caliper, in 60 healthy birth weight (10-90th percentile) term newborns without newborn complications and from pregnancies not affected by gestational diabetes, hypertension, dyslipidemia, smoking, amongst others.*	Large population representative samples.<br><br>International collaborative with standardized equipment, and assessment and measurement protocols.<br><br>Across the life course.

Key knowledge gaps & rationale for priority	Current evidence	Priorities & potential methods
	Infants: mean abdominal aortic IMT 0.618 mm (SD 0.050)[19]. *Measured by edge-detection software, in 814 term born infants (5-7 weeks old), drawn from a population-derived birth cohort, excluding those born preterm, those with congenital abnormalities, and those with significant neonatal illness.*<br><br>Adolescent males: mean abdominal aortic IMT 0.57 mm (SD 0.1),<br>Adolescent females: mean abdominal aortic IMT 0.55 mm (SD 0.1),<br>Young adult males: mean abdominal aortic IMT 0.68 mm (SD 0.1),<br>Young adult females: mean abdominal aortic IMT 0.64 mm (SD 0.1)[20].<br>*Measured by edge-detection software, in 606 adolescent and young adult participants in the Muscatine Offspring Cohort.*†	
**Feasibility and reproducibility in different ages and body sizes.**<br><br>Determine the age groups, and body sizes, in which abdominal aortic IMT is a feasible and reproducible technique.<br><br>Implications for clinical utility.	Feasibility – life course<br>Fetuses (30-36 weeks' gestation)[6,7], newborns [4], infants (12 months)[21], adolescents, young adults [20,22], *based on successful measurement in ~95% of participants, and in middle-aged adults >90% of participants* [23,24].<br><br>Feasibility – body size<br>Adolescents: BMI < 85th percentile, 100% success; BMI ≥ 85th and < 95th percentile, 97% success; BMI ≥ 95th percentile, 84.1% success [25].<br>Adults: BMI < 25 kg/m^2, 98.4% success; BMI ≥ 25 and < 30 kg/m^2, 97.5% success; BMI ≥ 30 kg/m^2, 84.8% success [25].<br><br>Reproducibility – life course<br>Newborns: intraobserver ICC = 0.86, coefficient of variation 2.6% for mean aortic IMT; intraobserver ICC = 0.93, coefficient of variation 2.1% for maximum aortic IMT; interobserver coefficient of variation 7.6% for mean aortic IMT; interobserver coefficient of variation 4.5% for maximum aortic IMT [8].<br>6-week old infants: inter-operator ICC = 0.84, intra-observer ICC = 0.90, inter-observer ICC = 0.92 [19].<br>18-month old children: intraobserver correlation coefficient = 0.88, interobserver correlation coefficient = 0.86 [6].<br>11 year old children: inter-observer ICC = 0.86, repeated scans (several months) ICC = 0.86 [1].	Determination of age ceiling for abdominal aortic IMT, and further development of fetal abdominal aortic IMT technique. Use of lower frequency ultrasound probes in adults, those with greater abdominal size, and during pregnancy for fetal abdominal aortic IMT.<br><br>Development and application of best practice imaging techniques for young children (2-3 years), in whom compliance with ultrasound testing generally is poor.

Key knowledge gaps & rationale for priority	Current evidence	Priorities & potential methods
	Adolescents: repeated scans‡ absolute difference = 0.053 mm (SD 0.042), coefficient of variation = 9.0% [25].<br>Young adults: repeated scans‡ absolute difference = 0.150 mm (SD 0.130), coefficient of variation = 21.6% [25].<br>Middle-aged adults: intra-operator coefficient of variation = 11%, inter-operator coefficient of variation = 10% [24].	
**Longitudinal tracking and normal rates of progression.**<br><br>Inform use of technique as surrogate endpoint.<br><br>Implications for clinical utility.	From 32 weeks' gestation (fetus) to 18 months of age: correlation coefficient = 0.48 in those identified with intrauterine growth restriction while *in utero* (n=22); no correlation in those with appropriate fetal growth (results not published)[6].<br><br>Cross-sectional analysis in adolescents and young adults indicates that the mean increase in abdominal aortic IMT with age is 0.10 mm/decade as compared to 0.04 mm/decade for carotid IMT [25].	Repeated measures in large population representative samples.<br><br>International collaborative with standardized equipment, and assessment and measurement protocols.<br><br>Across the life course.
**Clinical utility.**<br><br>Potential for use in risk stratification, and for assessing treatment benefit in high risk groups.	Currently no demonstrated clinical utility.	Focus on high risk individuals in age groups in which abdominal aortic IMT is of most pathophysiologic relevance (≤ 12 years of age).<br><br>Determine prediction of incident cardiovascular events in adults, relative to other risk markers, and both established and emerging cardiovascular risk factors.

ICC, intraclass correlation coefficient. * These fetal abdominal aortic IMT values are markedly higher than those reported elsewhere and in newborns. This may possibly be due to differences in equipment and measurement protocols, or to lesser distending pressure on the fetal aortic wall due to lower blood pressure [26-28]. † Estimated age-specific percentiles for abdominal aortic IMT and carotid IMT

in adolescents and young adults are shown in Figure 4. ‡ Mean time between scans was 38 days for combined adolescent and young adult groups.

Supplemental References

1. Jarvisalo, M.J.; Jartti, L.; Nanto-Salonen, K.; Irjala, K.; Rönnemaa, T.; Hartiala, J.J.; Celermajer, D.S.; Raitakari, O.T. Increased aortic intima-media thickness: a marker of preclinical atherosclerosis in high-risk children. *Circulation* **2001**, *104*, 2943-2947.
2. Harrington, J.; Peña, A.S.; Gent, R.; Hirte, C.; Couper, J. Aortic Intima Media Thickness is an Early Marker of Atherosclerosis in Children with Type 1 Diabetes Mellitus. *The Journal of Pediatrics* **2010**, *156*, 237-241.
3. Kallio, K.; Jokinen, E.; Saarinen, M.; Hamalainen, M.; Volanen, I.; Kaitosaari, T.; Ronnemaa, T.; Viikari, J.; Raitakari, O.T.; Simell, O. Arterial intima-media thickness, endothelial function, and apolipoproteins in adolescents frequently exposed to tobacco smoke. *Circ Cardiovasc Qual Outcomes* **2010**, *3*, 196-203.
4. Skilton, M.R.; Evans, N.; Griffiths, K.A.; Harmer, J.A.; Celermajer, D.S. Aortic wall thickness in newborns with intrauterine growth restriction. *Lancet* **2005**, *365*, 1484-1486.
5. Koklu, E.; Kurtoglu, S.; Akcakus, M.; Koklu, S.; Buyukkayhan, D.; Gumus, H.; Yikilmaz, A. Increased aortic intima-media thickness is related to lipid profile in newborns with intrauterine growth restriction. *Horm Res* **2006**, *65*, 269-275.
6. Cosmi, E.; Visentin, S.; Fanelli, T.; Mautone, A.J.; Zanardo, V. Aortic intima media thickness in fetuses and children with intrauterine growth restriction. *Obstet Gynecol* **2009**, *114*, 1109-1114.
7. Gomez-Roig, M.D.; Mazarico, E.; Valladares, E.; Guirado, L.; Fernandez-Arias, M.; Vela, A. Aortic intima-media thickness and aortic diameter in small for gestational age and growth restricted fetuses. *PLoS One* **2015**, *10*, e0126842.

8. Stergiotou, I.; Crispi, F.; Valenzuela-Alcaraz, B.; Cruz-Lemini, M.; Bijnens, B.; Gratacos, E. Aortic and carotid intima-media thickness in term small-for-gestational-age newborns and relationship with prenatal signs of severity. *Ultrasound Obstet Gynecol* **2014**, *43*, 625-631.

9. Cruz-Lemini, M.; Crispi, F.; Valenzuela-Alcaraz, B.; Figueras, F.; Gómez, O.; Sitges, M.; Bijnens, B.; Gratacós, E. A fetal cardiovascular score to predict infant hypertension and arterial remodeling in intrauterine growth restriction. *Am J Obstet Gynecol* **2014**, *210*, 552.e551–552.e522.

10. Koklu, E.; Akcakus, M.; Kurtoglu, S.; Koklu, S.; Yikilmaz, A.; Coskun, A.; Gunes, T. Aortic intima-media thickness and lipid profile in macrosomic newborns. *Eur J Pediatr* **2007**, *166*, 333-338.

11. McCloskey, K.; Burgner, D.; Carlin, J.B.; Skilton, M.R.; Cheung, M.; Dwyer, T.; Vuillermin, P.; Ponsonby, A.L.; BIS investigator group. Infant adiposity at birth and early postnatal weight gain predict increased aortic intima-media thickness at 6 weeks of age: a population-derived cohort study. *Clin Sci (Lond)* **2016**, *130*, 443-450.

12. McCloskey, K.; Vuillermin, P.; Carlin, J.B.; Cheung, M.; Skilton, M.R.; Tang, M.L.; Allen, K.; Gilbert, G.L.; Ranganathan, S.; Collier, F., et al. Perinatal microbial exposure may influence aortic intima-media thickness in early infancy. *Int J Epidemiol* **2017**, *46*, 209-218.

13. Volanen, I.; Jarvisalo, M.J.; Vainionpaa, R.; Arffman, M.; Kallio, K.; Angle, S.; Ronnemaa, T.; Viikari, J.; Marniemi, J.; Raitakari, O.T., et al. Increased aortic intima-media thickness in 11-year-old healthy children with persistent Chlamydia pneumoniae seropositivity. *Arterioscler Thromb Vasc Biol* **2006**, *26*, 649-655.

14. Begg, L.M.; Palma-Dias, R.; Wang, J.; Chin-Dusting, J.P.F.; Skilton, M.R. Maternal adiposity and newborn vascular health. *Arch Dis Child Fetal Neonatal Ed* **2013**, *98*, F279-F280.

15. Lo Vasco, V.R.; Salmaso, R.; Zanardo, V.; Businaro, R.; Visentin, S.; Trevisanuto, D.; Cosmi, E. Fetal aorta wall inflammation in ultrasound-detected aortic intima/media thickness and growth retardation. *Journal of reproductive immunology* **2011**, *91*, 103-107.

16. Pignoli, P.; Tremoli, E.; Poli, A.; Oreste, P.; Paoletti, R. Intimal plus medial thickness of the arterial wall: a direct measurement with ultrasound imaging. *Circulation* **1986**, *74*, 1399-1406.

17. Hondappanavar, A.; Sodhi, K.S.; Dutta, S.; Saxena, A.K.; Khandelwal, N. Quantitative ultrasound measurement of intima-media thickness of abdominal aorta and common carotid arteries in normal term newborns. *Pediatric cardiology* **2013**, *34*, 364-369.

18. Koklu, E.; Kurtoglu, S.; Akcakus, M.; Yikilmaz, A.; Coskun, A.; Gunes, T. Intima-media thickness of the abdominal aorta of neonate with different gestational ages. *J Clin Ultrasound* **2007**.

19. McCloskey, K.; Ponsonby, A.L.; Carlin, J.B.; Jachno, K.; Cheung, M.; Skilton, M.R.; Koleff, J.; Vuillermin, P.; Burgner, D.; Barwon Infant Study investigator group. Reproducibility of aortic intima-media thickness in infants using edge-detection software and manual caliper measurements. *Cardiovascular ultrasound* **2014**, *12*, 18.

20. Dawson, J.D.; Sonka, M.; Blecha, M.B.; Lin, W.; Davis, P.H. Risk factors associated with aortic and carotid intima-media thickness in adolescents and young adults: the Muscatine Offspring Study. *J Am Coll Cardiol* **2009**, *53*, 2273-2279.

21. Kizirian, N.V.; Kong, Y.; Muirhead, R.; Brodie, S.; Garnett, S.P.; Petocz, P.; Sim, K.A.; Celermajer, D.S.; Louie, J.C.; Markovic, T.P., et al. Effects of a low-glycemic index diet

during pregnancy on offspring growth, body composition, and vascular health: a pilot randomized controlled trial. *Am J Clin Nutr* **2016**, *103*, 1073-1082.

22. Pahkala, K.; Hietalampi, H.; Laitinen, T.T.; Viikari, J.S.; Ronnemaa, T.; Niinikoski, H.; Lagstrom, H.; Talvia, S.; Jula, A.; Heinonen, O.J., et al. Ideal cardiovascular health in adolescence: effect of lifestyle intervention and association with vascular intima-media thickness and elasticity (the Special Turku Coronary Risk Factor Intervention Project for Children [STRIP] study). *Circulation* **2013**, *127*, 2088-2096.
23. Koc, A.S.; Sumbul, H.E. Increased aortic intima-media thickness may be used to detect macrovascular complications in adult type II diabetes mellitus patients. *Cardiovascular ultrasound* **2018**, *16*, 8.
24. Astrand, H.; Sandgren, T.; Ahlgren, A.R.; Lanne, T. Noninvasive ultrasound measurements of aortic intima-media thickness: implications for in vivo study of aortic wall stress. *J Vasc Surg* **2003**, *37*, 1270-1276.
25. Davis, P.H.; Dawson, J.D.; Blecha, M.B.; Mastbergen, R.K.; Sonka, M. Measurement of aortic intimal-medial thickness in adolescents and young adults. *Ultrasound Med Biol* **2010**, *36*, 560-565.
26. Struijk, P.C.; Mathews, V.J.; Loupas, T.; Stewart, P.A.; Clark, E.B.; Steegers, E.A.; Wladimiroff, J.W. Blood pressure estimation in the human fetal descending aorta. *Ultrasound Obstet Gynecol* **2008**, *32*, 673-681.
27. Dionne, J.M.; Abitbol, C.L.; Flynn, J.T. Hypertension in infancy: diagnosis, management and outcome. *Pediatr Nephrol* **2012**, *27*, 17-32.
28. Polak, J.F.; Johnson, C.; Harrington, A.; Wong, Q.; O'Leary, D.H.; Burke, G.; Yanez, N.D. Changes in carotid intima-media thickness during the cardiac cycle: the Multi-

Ethnic Study of Atherosclerosis. *Journal of the American Heart Association* **2012**, *1*, e001420.
